# What to Observe at the Bedside and after Death in Medical Cases

**Published:** 1853-07

**Authors:** 


### EDITORIAL.

*What to observe at the bed-side and after death in Medical Cases.* Published under the authority of the London Medical Society of Observation. Philadelphia: Blanchard & Lea, 1853.

This work was designed for the purpose of giving uniformity to the order of the clinical observations of the members of the society. It suggests to the physician the different subjects to be investigated and organs to be examined, as well as the order in which each should be taken up. These are all particulars in which many physicians are deficient, and on the account of which their observations lose much of their value.

For sale by A. H. & C. Burley. J.

